# ERRATUM: Nursing workload: is it a predictor of healthcare associated infection in intensive care unit?

**DOI:** 10.1590/1980-220X-REEUSP-2024-ER06en

**Published:** 2024-09-27

In the article Nursing workload: is it a predictor of healthcare associated infection in intensive care unit?, with the DOI: https://doi.org/10.1590/S0080-623420150000700006, published by the journal: “Revista da Escola de Enfermagem da USP, volume 49 (spe) of 2015, p. 35–41, on page 41:

**Now read:**

## Financial support

This study was financed in part by the Coordenação de Aperfeiçoamento de Pessoal de Nível Superior – Brasil (CAPES) – Finance Code 001.

