# Supplementary material for: ERRATUM: Nursing workload: is it a predictor of healthcare associated infection in intensive care unit?
Source: Rev Esc Enferm USP. 2024 Sep 27;58:e2024ER06. doi: 10.1590/1980-220X-REEUSP-2024-ER06en (PMC11441790; doi:10.1590/1980-220X-REEUSP-2024-ER06en)
Supplement: Translation

# Translation: ERRATA: Carga de trabalho de enfermagem: preditor de infecção relacionada à assistência à saúde na terapia intensiva?

**DOI:** 10.1590/1980-220X-REEUSP-2024-ER06pt

No artigo Carga de trabalho de enfermagem: preditor de infecção relacionada à assistência à saúde na terapia intensiva?, com número de DOI: https://doi.org/10.1590/S0080-623420150000700006, publicado na Revista da Escola de Enfermagem da USP, volume 49 (spe) de 2015, p. 36–42, na página 42:

**Leia-se:**

## Apoio financeiro

O presente trabalho foi realizado com apoio da Coordenação e Aperfeiçoamento de Pessoal de Nível Superior – Brasil (CAPES) – Código de Financiamento 001.
